# Oral Floor and Gingival Metastasis of Cholangiocarcinoma: A Case Report and Review of the Literature

**DOI:** 10.1155/2014/712912

**Published:** 2014-05-07

**Authors:** Yukihiro Nakanishi, Bo Xu, Charles LeVea

**Affiliations:** ^1^Department of Pathology, Roswell Park Cancer Institute, Elm and Carlton Streets, Buffalo, NY 14263, USA; ^2^IU Health Pathology Laboratory, Department of Pathology, School of Medicine, Indiana University, 350 W. 11th Street, Room 4070, Indianapolis, IN 46202, USA

## Abstract

The oral cavity is very unusual site of metastases even though wide spread metastatic disease may be present. The most common primary sites that metastasize to the oral cavity are lung, kidney, breast, and hepatocellular carcinoma. We present a rare case of a 77-year-old Caucasian female with metastasis from a cholangiocarcinoma to the oral floor contiguous with lingual gingival mucosa. The patient presented with left sided rib pain. A CT scan of the chest, abdomen, and pelvis showed multiple pulmonary nodules and a single dominant mass in the right lobe of the liver. This tumor was 6.5 cm with multiple satellite lesions surrounding it. The liver biopsy was diagnostic of a moderately to poorly differentiated adenocarcinoma, consistent with a primary cholangiocarcinoma. After undergoing one cycle of gemcitabine chemotherapy, the patient noticed an extremely rapidly growing mass involving her right lower gingiva and the entire right floor of her mouth. The biopsy of that mass also showed a moderately to poorly differentiated adenocarcinoma. The gingival tumor had a similar cytomorphology and immunophenotype as her cholangiocarcinoma. Therefore, an unusual site for metastatic cholangiocarcinoma was confirmed.

## 1. Introduction

The oral cavity is very unusual site of metastasis accounting for only 1-2% of oral malignancy [1, 2]. The jawbones, particularly the mandible, were more commonly affected than the soft tissues of oral cavity [1–3]. The most common primary sites able to metastasize to the oral cavity are lung, kidney, breast, and hepatocellular carcinoma [1–4]. In this paper, we report a rare case of metastasis to the oral floor contiguous with lingual buccal mucosa from a cholangiocarcinoma.

## 2. Case Report

**Figure 1:**
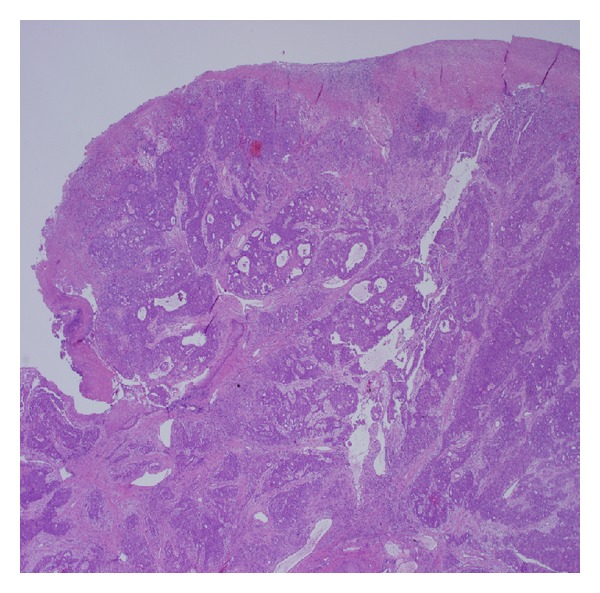
Low power view of the excised pedunculated gingival mass shows a polypoid tumor consisting of irregular nests of tumor cells.

**Figure 2:**
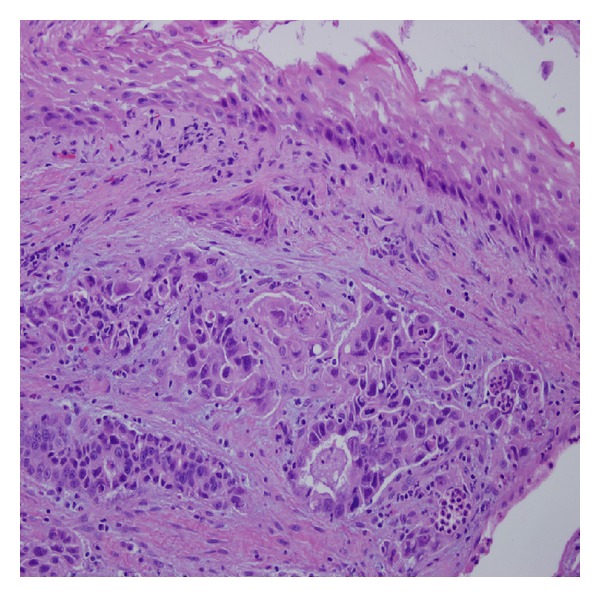
High power view of the excised polypoid gingival mass shows a moderately to poorly differentiated adenocarcinoma focally covered with nonneoplastic squamous mucosa.

The patient was a 77-year-old Caucasian female with a surgical history of tonsillectomy with adenectomy, appendectomy, partial gastrectomy for bleeding secondary to peptic ulcer disease, ovarian cyst, partial knee replacement, and cataract removal. The patient presented complaining of left sided rib pain that started in November, 2012. This was sudden in onset and was worse with coughing and movement. A chest X-ray showed some vague nodular densities in both lung fields. A CT scan of the chest, abdomen, and pelvis in mid-January, 2013, showed multiple pulmonary nodules and a single dominant mass in the right lobe of the liver, which was 6.5 cm with multiple satellite lesions. Bulky retroperitoneum and upper abdominal lymphadenopathy were present. Laboratory data showed increased serum levels of the tumor marker, CA19-9 at 560.1 (0.0–35.0 U/mL). Upper and lower gastrointestinal endoscopy did not show any malignancies. A liver biopsy was performed and reviewed at Roswell Park Cancer Institute. The diagnosis was a moderately to poorly differentiated adenocarcinoma. The tumor cells are positive for cytokeratin 7, cytokeratin 20, and CA19-9, and they are negative for TTF-1, CA125, thrombomodulin, and mammaglobin. The patient was diagnosed with a primary cholangiocarcinoma based on laboratory data and radiologic and pathologic findings. After undergoing one cycle of gemcitabine chemotherapy, the patient noticed an extremely rapidly growing mass involving her right lower gingiva and the entire right floor of mouth. This mass grew as a discrete pedunculated lesion between the teeth and within the lingual gingiva, causing significant trismus. Histologic examination showed a moderately to poorly differentiated adenocarcinoma, consistent with metastatic cholangiocarcinoma (Figures 1 and 2). The polypoid tumor was ulcerated through the overlying nonneoplastic squamous mucosa. The tumor cells are diffusely and strongly positive for cytokeratin 20, villin, and focally positive for CDX2, CA19-9, and CK7. The tumor cells share a similar cytomorphology and immunophenotype with the liver tumor. The patient was diagnosed with metastatic cholangiocarcinoma to the oral cavity based on the clinical and pathological findings. The patient decided not to receive any further treatment and chose hospice care.

## 3. Discussion

The oral cavity is very unusual site of metastasis. Metastatic disease accounts for about 1-2% of oral malignancy [1, 2]. Hirshberg et al. have reported that the jawbones, particularly the mandible, were reported to be more frequently affected than the oral soft tissues in a ratio of 2 to 1. In the oral soft tissues, the gingiva was the most commonly affected site [1]. Hirshberg et al. have reported that the major primary sites that may show oral cavity metastases were lung, kidney, liver, prostate, breast, uterus, ovaries, cervix, colon, and rectum based on the analysis of 673 cases with oral metastases [1]. Van der Waal et al. have reported that the most common metastases were from breast, lung, kidney, and prostate in that order of frequency based on the analysis of 24 cases with oral metastases [2]. In Korean studies, the most site of origin was reported to be from hepatocellular carcinoma [3, 4]. These differences may be caused by a relatively high incidence of hepatocellular carcinoma in Korea. Floor of mouth and gingival metastasis from cholangiocarcinoma are extremely rare. To our knowledge, this is the first case report of cholangiocarcinoma metastasized to the floor of mouth contiguous with lingual gingival mucosa. Several intraoral primary tumors may mimic metastatic tumors, for instance, primary ductal carcinoma of the salivary gland versus metastatic breast carcinoma, primary intraoral clear cell tumor of the salivary gland versus metastatic renal cell carcinoma, and primary squamous cell carcinoma of the oral cavity versus metastatic squamous cell carcinoma of the lung.

Oral metastases are usually evidence of a widespread disease, as occurred in this case, and indicate a grave prognosis [5]. However, in nearly 30% of patients with oral metastases, the metastatic lesion in the oral region is the first indication of undiscovered malignancy at a distant site [5]. The time from the appearance of the metastasis to death is usually a few months [6].

Oral metastasis may be present in the soft tissues or in the jawbones [5]. There are more published cases of jawbone metastases than metastases in oral soft tissues [6]. In the oral soft tissues, the gingiva is the most commonly affected site, followed by the tongue [7]. As for the pathogenesis of gingival metastasis, Hirshberg proposes that inflammation helps attract the metastatic tumor cells to the gingiva [5]. Malignant tumor cells may get entrapped in the rich capillary network of chronically inflamed gingiva. Proliferating capillaries have a fragmented basement membrane, are leaky, and, therefore, are more penetrable by malignant tumor cells than mature vessels [8]. The tongue, the second most common site of metastasis, is well-vascularised and may have the potential to attract metastatic tumor cells [5].
